# Progression from Sustained BK Viruria to Sustained BK Viremia with Immunosuppression Reduction Is Not Associated with Changes in the Noncoding Control Region of the BK Virus Genome

**DOI:** 10.1155/2012/761283

**Published:** 2012-06-04

**Authors:** Imran A. Memon, Bijal A. Parikh, Monique Gaudreault-Keener, Rebecca Skelton, Gregory A. Storch, Daniel C. Brennan

**Affiliations:** ^1^Department of Pediatrics, Washington University School of Medicine, St. Louis, MO 63110, USA; ^2^Department of Internal Medicine, Washington University School of Medicine, St. Louis, MO 63110, USA; ^3^Department of Pathology, Washington University School of Medicine, St. Louis, MO 63110, USA; ^4^Barnes-Jewish Hospital, Washington University School of Medicine, St. Louis, MO 63110, USA

## Abstract

Changes in the BK virus archetypal noncoding control region (NCCR) have been associated with BK-virus-associated nephropathy (BKVAN). Whether sustained viremia, a surrogate for BKVAN, is associated with significant changes in the BK-NCCR is unknown. We performed PCR amplification and sequencing of (1) stored urine and (2) plasma samples from the time of peak viremia from 11 patients with sustained viremia who participated in a 200-patient clinical trial. The antimetabolite was withdrawn for BK viremia and reduction of the calcineurin inhibitor for sustained BK viremia. DNA sequencing from the 11 patients with sustained viremia revealed 8 insertions, 16 transversions, 3 deletions, and 17 transitions. None were deemed significant. No patient developed clinically evident BKVAN. Our data support, at a genomic level, the effectiveness of reduction of immunosuppression for prevention of progression from viremia to BKVAN.

## 1. Introduction

Human polyomavirus and associated infection were first identified in a urine sample of a transplant recipient with ureteric stenosis in 1971 [1]. BK-virus-associated nephropathy (BKVAN) was first recognized in kidney transplant recipients in 1995 [2]. The sequence of histopathologic events is characterized by tubular necrosis, mixed interstitial inflammatory infiltration, and finally, scarring and fibrosis of the renal interstitium [3]. BKV nephropathy currently affects 1–7% of recipients and has been associated with a 10–100% graft loss rate depending on the severity of histological involvement [4, 5]. It is likely that the last stage of an unchecked BKV infection begins as asymptomatic viruria, progresses to sustained viremia, possibly associated with subclinical nephritis, and culminates in overt nephropathy. Thus, sustained BKV detected in plasma predicts progression to BKV-associated interstitial nephritis, or nephropathy [6].

JC virus and SV40 virus are other members of the family Polyomaviridae [7] and share 75% and 70% homology, respectively, with BKV. They have three general transcription regions: the noncoding control region (NCCR); the early coding region, which codes for the small and large T antigens; and the late coding region, which codes for the viral capsid proteins VP-1, VP-2, and VP-3 [8]. A fourth region, the agnogene, encodes for the agnoprotein. The agnoprotein has recently been thought to play a role in replicative life-cycle of polyomaviruses and also in the dysregulation of cellular processes such as cell cycle control and DNA repair [9, 10].

**Figure 1:**
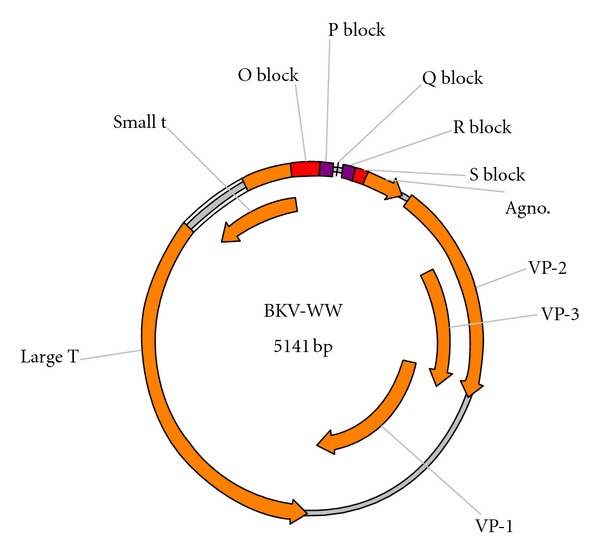
Diagram showing the archetypal wild type BK virus structure. The O, P, Q, and R blocks represent the noncontrol coding region (NCCR).

The NCCR consists of the origin of replication [11] and the transcription control region and is arbitrarily classified into O, P, Q, R, and S regions as an aid to visualize genetic rearrangements [12, 13] (Figure 1). These sites control the expression of the early genes, late genes, and the agnoprotein [13, 14]. The NCCR also contains a number of transcription factor binding sites involved in the regulation of viral genes. These include Sp-1 (specificity protein 1), NF-1 (nuclear factor 1), and CRE (cyclic-AMP response element). Additionally, promoter-enhancing sequences that control viral replication and regulate cellular oncogenes (c-*myc*) and the *p53 *tumor suppressor gene are found within the BKV NCCR [11].

**Figure 2:**
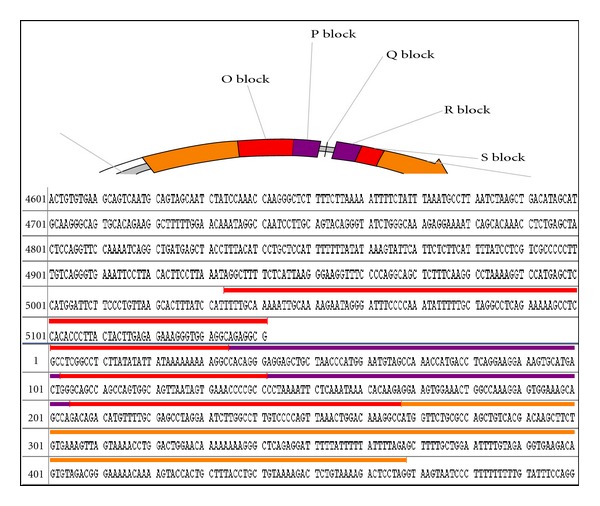
Diagram showing the noncontrol coding region (NCCR) region and describing the nucleotide sequence of the O, P, Q, R, and S blocks in different colors.

BKV contains a fixed number of nucleotides per NCCR region. The archetypal strain of BKV, WW, is described as O142, P63, Q39, R63, and S63, where block O consists of 142 base pairs, block P with 63, and so on (Figure 2). Any deviation from the archetypal sequence and/or structural formula represents a genetic variation in the NCCR region.

Although BKV strains are genotypically classified based on polymorphisms in the VP-1 region (genotypes I–IV), based on the NCCR structure, BKV variants are classified either as archetype or as rearranged forms [15]. Therefore, BKV variants with rearranged NCCRs are not considered unique strains. Initially, it was proposed that NCCR rearrangements arose as an adaptation of the virus to cell culture. With the application of polymerase chain reaction (PCR) technology, one can amplify and sequence the naturally occurring NCCR from clinical specimens, to determine whether a rearrangement has occurred. The archetypal BK-NCCR structure is the most common form found in the urine [16]. It has been suggested that in permissive cells the virus replicates and gives rise to rearranged enhancers [14, 17], which permit viral species to adjust to transcription conditions within the cell [18]. This might explain the occurrence of BKV variants in specific tissues and under different clinical conditions; however, the mechanisms or pressures, such as immunosuppression, regulating the rearrangement events are unclear. Although associations of rearranged viral strains with greater replication propensity have been described, most publications are based on very small data sets [5, 7]. Relationships between genetic rearrangement, viral genotype, tissue tropism, geographic distribution, pathogenicity, and progression of disease remain inadequately studied.

In addition to genomic rearrangements, genetic variation in the BK-NCCR can arise from point mutations, deletions, and duplications of the basic sequence elements and can affect the transcriptional, transformational, and replicative potential of the BKV variant [13]. Functional promoter analysis and computational approaches have identified a number of transcription factor binding sites within the BK-NCCRs, such as a CRE within the P block, two SP-1 binding elements within the Q and R blocks, and four NF-1 binding sites within the P, Q, R, and S blocks [13]. Although the coding region of the BKV genome is highly conserved, minimal variations in the late region encoding the major viral capsid protein VP-1 allow BKV to be divided into four genotypes (I–IV).

A variety of genomic rearrangements have been detected in the NCCR of the related human polyomavirus, for example, JC virus taken from the brain tissue of patients with progressive multifocal leukoencephalopathy (PML). These rearrangements seem to be crucial in permitting viral replication in the brain and the lysis of oligodendrocytes [11]. This is gaining increased clinical relevance given the increased incidence and Black Box warnings regarding progressive multifocal leukoencephalopathy (PML) associated with the use of mycophenolate mofetil and Belatacept.

The association of BKV subtypes with clinical conditions only recently has been described [19]. One study demonstrated that BKV subtype I was predominant in urine samples from bone marrow transplant recipients with hemorrhagic cystitis. Other potential viral determinants of hemorrhagic cystitis included overrepresentation of cytosine (C) to guanosine (G) mutations in the NCCR SP-1 binding site, limited to 7 of 13 patients with hemorrhagic cystitis. However, these alterations were not associated with increased urinary BKV loads, arguing against the pathogenicity of these variants [20].

A number of BKV-DNA sequence variants have been described in the form of point mutations (transitions and transversions), duplications, deletions, and rearrangements localized to the NCCR of the viral DNA in renal transplant patients, but they were not associated with disease. Boldorini et al., however, reported the presence of NCCR rearrangement in 3 of 5 (60%) biopsies with BKVAN [11]. Although they concluded that the NCCR rearrangements did not necessarily correlate with the stage of BKVAN, the presence of rearrangements in the majority of BKVAN samples suggests a pathogenic role of rearrangement in progression of BK virus infection. Similarly, Olsen et al. reported an incidence of rearrangement of NCCR region in 50% of patients with BKVAN [21]. A study of 30 renal transplant recipients with BK viremia did not show any changes in the NCCR region in the urine or plasma samples with high titer viruria or viremia but none of these recipients developed BKVAN [22].

Thus, studies evaluating the rearrangement or mutation in the NCCR as a factor in the pathogenesis and virulence of BK virus have involved small numbers of patients and have not been definitive. The purpose of this study was to determine whether the presence of NCCR rearrangements or mutations in the urine and blood of patients with sustained BK viremia, a surrogate of BKVAN, was associated with progression from BK viruria.

## 2. Materials and Methods

### 2.1. Study Design

From December 2000 to October 2002, 200 *de novo *kidney transplant recipients were enrolled in a prospective open label trial and randomized to receive tacrolimus or cyclosporine in a 2 : 1 block-design fashion stratified by race and gender as previously described [23]. Recipients were prospectively monitored for the development of BK viruria, viremia, and nephropathy. Identification of BK viremia triggered discontinuation of the antimetabolite component of the immunosuppressive regimen upon detection of BK-viremia and reduction in the calcineurin component upon detection of sustained viremia to prevent progression of viremia to nephropathy. Clinical data and frozen plasma and DNA samples collected during 1-year of follow-up were analyzed based on the development of BK viruria, any viremia, and sustained viremia, defined as ≥2 consecutive BKV positive plasma samples spanning ≥3 weeks. For the purposes of this study we selected samples from the eleven patients with sustained viremia (as defined here in after) around the time of peak viral load in blood. The Washington University Human Studies Committee approved the study, and all patients gave informed consent for the initial and follow-up studies.

### 2.2. Sample Collection and Preparation

Samples consisted of undiluted whole blood, plasma, and urine samples collected before -transplant, weekly for 16 weeks, and at months 5, 6, 9, and 12. Samples were stored at −80°C. We identified 11/200 (6%) of patients with sustained BK viremia and high urine viral load in our cohort. We selected one urine and two plasma samples from each patient near the time of highest blood viral load as viremia correlates with tissue invasive disease.

### 2.3. BKV PCR Detection

Established qualitative PCR for detection of BKV DNA was used as previously described and performed in a diagnostic clinical laboratory [16, 23]. Briefly, DNA was extracted and purified from urine and plasma using QIAamp spin columns (QIAGEN Inc., Valencia, CA) according to the manufacturer's instructions. Extracted DNA was tested for BK virus DNA by real-time PCR using the Light Cycler System (Roche Diagnostics Corporation, Indianapolis, IN) using the primers, Pep-1 AGT CTT TAG GGT CTT CTA CC and Pep-2 GGT GCC AAC CTA TGG AAC AG, which amplify a 176-basepair segment of the BK virus large T-antigen gene [24]. BK virus-specific hybridization probes 5-TTG CCA TGA AGA TAT GTT TGC CAG TGA TGA FITC-3 and 5-LCRed640 GAA GCA ACA GCA GAT TCT CA_3 (TIB Molbiol LLC, Adelphia, NJ) were used for detection. These probes were designed to detect BK virus without detection of the related polyoma viruses, JC virus and SV-40. Reactions were carried out in a 20 *μ*L volume that included 2 *μ*L of Roche Light Cycler FastStart DNA Hybridization Probe 10× reaction mix and 2 *μ*L of sample DNA. The final concentrations of other components were 3.5 mM MgCl_2_, 0.5 *μ*M of each primer and 0.2 *μ*M of each probe. The reaction program consisted of denaturation for 7 minutes at 95°C followed by 45 cycles of denaturation at 95°C instantaneous, annealing at 52°C for 10 seconds, and extension at 75°C for 7 seconds. Each reaction included positive controls consisting of 1000 copies of BK virus plasmid DNA (ATCC 45026) obtained from the American Type Culture Collection (ATCC, Manassas, VA) and negative controls for amplification and DNA preparation. The sensitivity of the qualitative assay was 20–50 BK virus DNA copies per reaction.

### 2.4. BKV PCR Quantification

BK-positive samples were quantified by repeat real-time PCR analysis of aliquots of extracted DNA that had been frozen at −80°C, alongside a standard curve of control BKV DNA (2 × 10^8^, 2 × 10^6^, 2 × 10^4^, and 2 × 10^2^). The standards were prepared from a plasmid (pBKV [35-1]) that contains the entire linearized BKV genome (ATCC 45026) and was quantified by spectrophotometry. The coefficient of variability for the quantitative BKV LightCycler assay in our laboratory was less than 6%. The lower limit of sensitivity of the quantitative PCR assay is 10000 copies/mL.

### 2.5. Amplification and Sequencing of the BKV Noncoding Control Region

Nested PCR was carried out using the following protocol [25]. The first round PCR with primers NCCR 722R (5′ to 3′) TTTCCCGTCTACACTGTCTTCACC and NCCR 128F (5′ to 3′) CCCAGGCAGCTCTTTCAAGG amplified a 596-bp fragment of BKV, including the NCCR. The nested PCR reaction used primers NCCR 596 R (5′ to 3′) TGACAGCTGGCGCAGAACC and NCCR 164F (5′ to 3′) GCTCCATGGATTCTTCCCTGTTAAGC which amplified a 432 bp fragment of the previous PCR product.

The DNA amplification reactions were carried out in 100 *μ*L volumes with 10 *μ*L of patient or control DNA, 1.25 *μ*L of Taq DNA polymerase (Invitrogen, Carlsbad, CA), 1.25 *μ*L of each primer, and 5 *μ*L of PCR buffer including deoxynucleoside triphosphates. The DNA amplification reactions for the nested PCR were carried out in 100 *μ*L volumes with 5 *μ*L of from the initial reaction product, 1.875 *μ*L of Taq DNA polymerase (Invitrogen, Carlsbad, CA), 1.875 *μ*L of each primer, and 7.5 *μ*L of PCR buffer including deoxynucleoside triphosphates.

Thermal cycling comprised an initial hot start at 94°C for 3 minutes followed by 30 cycles of 68°C for 45 seconds and the final step at 68°C for 1 minute. The PCR products were visualized on a UV transilluminator, followed by gel electrophoresis in 2% agarose gels containing ethidium bromide. The PCR products were purified using a MinElute PCR purification kit (Qiagen, Valencia, CA).

Single-band PCR products of both strands were sequenced using BigDye Terminator DNA-sequencing techniques (Applied Biosystems, Foster City, CA) using both the forward and reverse primers of the nested PCR reaction and the sequencing data were analyzed using Vector NTI software (Invitrogen, Carlsbad, CA) to obtain a consensus sequence using both strands.

## 3. Results and Discussion

Our prior studies showed that BK virus DNA was detected in urine samples from 70 recipients (35% of the 198 with samples available for analysis) [23]. The estimated incidence of BK viruria through 365 days after -transplant was 35%. The baseline demographic characteristics did not affect the incidence of BK viruria. BK viremia was detected in 23 patients. BK viremia never occurred in the absence of viruria and followed or was contemporaneous with BK viruria. The overall estimated incidence of viremia was 12% through 365 days. Sustained BK viremia was detected in 11 (6%) patients. No clinically evident BKVAN was seen by 12 months, and none had been seen at the last date of observation.

We anticipated a total of 33 samples with BK viruria available for analysis. However, one patient had insufficient DNA in the urine sample to allow for further amplification, and we were not able to amplify the BK virus in one of the plasma samples in two patients and in both plasma samples in one patient. Thus, we had 28 complete NCCR sequences to analyze.

Among the 11 patients with sustained viremia, the mean viral load in the plasma samples was 5.08 log_10_ copies/mL (range 2.5 Log_10_–5.90 log_10_ copies/mL), with a median of 5.49 log_10_ copies/mL. The mean viral load in the urine samples was 10.3 log_10_ copies/mL (range 8.32 log_10_–12.02 log_10_ copies/mL), with a median of 10.17 log_10_ copies/mL.

**Figure 3:**
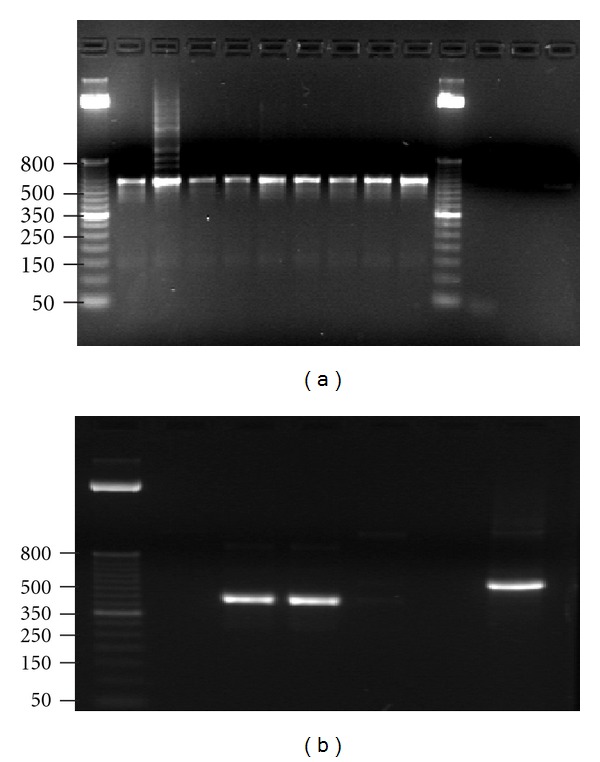
Gel electrophoresis showing the DNA-ladder on each end with the all urine samples (a) and plasma samples (b) with the DNA-ladder on the left side showing similarity between of NCCR region size in each sample.

Given the magnitude of our viral load we expected to find significant rearrangements, that is, the addition of whole blocks in the NCCR regions of the amplification product. We initially evaluated the size of the product and compared this to our control. We did not find any significant change when compared to control (ATCC 45026). Only two of our plasma samples showed two different bands on gel electrophoresis which were only 25 bp different from the control suggesting that there might be smaller mutations in our samples (Figure 3). Thus, we further examined our products after DNA sequencing to assess for small changes in the sequence. We compared our sequence results with the wild type NCCR reported in the literature [18].

We only found small changes in the BK virus genome (Table 1). In total, we found 8 insertions, 16 transversions, 3 deletions, and 17 transitions. We defined transitions as a pyrimidine nucleotide replacing a pyrimidine and a transversions as a replacement of pyrimidine with a purine or vice versa. Deletion and insertion were defined as a removal or addition of a nucleotide. However, no significant rearrangements or sequence variations such as duplications or inversions were identified. No changes were noted in the aforementioned Sp-1, CRE, or NF-1 sites.

The pathogenesis of BK virus infection is not clearly understood. Based on observation studies, it is thought that BK virus infection occurs due to the failure in the balance between BK virus replication and natural immunological control of that process resulting in BKVAN. In addition to host factors, it is hypothesized that various viral factors contribute to the pathogenesis of BKVAN. To date all studies looking at these factors have been uncontrolled and small and only suggestive of the possibility of a viral determinant of BKVAN [26].

It has been reported that BKV variants with rearranged NCCR are present in immunocompromised patients with kidney transplants [18, 21, 26]. In a retrospective analysis of matched urine and plasma samples from patients with any BK viremia of which 70 had BKVAN, Gosert et al. reported that rearranged NCCR variant BKV replaced the wild type in 24% of kidney transplant patients with persistent BK viremia and in 50% of patients with BKVAN [5]. BK virus with rearranged NCCR had a 20-fold higher median viral load and higher probability of histologically confirmed disease when compared to wild type NCCR BKV. Rearranged NCCR was detectable in patients with peak viral load of 6.5 log10 genome equivalents(geq)/mL for <4 months, whereas a 100-fold lower viremia required >7 times longer times suggesting that the duration and higher peak viral load favored the change from the wild type, ww-NCCR, to rearranged, rr-NCCR, as a majority species. They confirmed the role of rearrangements in the NCCR and polyomavirus nephropathy in a prospective cohort of 73 plasma samples from 227 kidney transplant recipients of which 39 had BKVAN. The role of rearranged NCCR was further confirmed in elegant in vitro studies using a novel bidirectional reporter replicon that mimics polyoma replication.

Their patients were not in a clinical trial and no systematic intervention such as reduction in immunosuppression was used for clinical management of viremia. Our patients were in a clinical trial, and since we promptly reduced immunosuppression upon detection of viremia, none of our patients developed very high viral loads (peak viral load of 5.90 log10 copies/mL) for more than a month. Thus, our study supports the findings of Gosert et al. and explains the absence of rearranged NCCR in our samples.

Bressollette-Bodin et al. reported results showing that NCCR mutation or rearrangement was not present in patients with viremia or viruria [22]. It is unclear from their data whether they had sustained viremia or viruria. The number of samples with high titer viremia and viruria was lower than that of our patients and BKVAN was not observed. It is unclear whether this was due to the fact that their immunosuppression regimen was different than ours including a prednisone-free regimen and their follow-up was for only a year. Thus it cannot be concluded if and when their patients developed BKVAN and whether the NCCR mutation may have occurred.

We found only minor changes in the NCCR region associated with sustained viremia. We did not observe any clinically evident BKV nephropathy. Thus, we could not determine whether changes in the BK-NCCR region might be associated with progression from sustained BK-viremia to BKVAN. Significantly, the peak viral load in our patients was lower than that in the study by Gosert et al. perhaps as a consequence of our clinical management of immunosuppressive reduction upon detection of BK-viremia [5]. Our interventional strategy of withdrawal of the antimetabolite and further reduction in immunosuppression with persistence of sustained viremia may have removed the selective pressure that would have led to rearrangements and subsequent development of BKVAN. There may also be a threshold of viral load that may determine tissue invasive disease, that is, a higher load being suggestive of aggressive disease with mutation or pathogenetic rearrangements in the NCCR.

The major limitation of this study is that there was no clinically evident BKVN; hence it cannot be assumed that if it had occurred, it may have resulted from changes in the NCCR. Given the serious impact of BK viremia, we felt that it was unethical simply to follow the patients without intervention. Our strategy was successful in the short and long term and resulted in excellent clinical outcomes [16, 23, 27]. The current study provides translational and mechanistic support, at a genomic level, for our interventional approach. Although the sample size, *n* = 11, of patients with sustained viremia, a surrogate of BKVAN, is small, it is the largest such assessment to date looking at whether NCCR arrangements are associated with sustained viremia.

## 4. Conclusion

Sustained viremia in our study in association with significant immunosuppression withdrawal upon detection of BK viremia was not associated with rearrangement and/or sequence variation in the NCCR region of the BK virus. Our findings do not exclude the possibility of an association between transition from sustained BK viremia to BKVAN based on NCCR rearrangement or sequence variation. Our data, however, support the effectiveness of a clinical interventional approach of withdrawal and reduction of immunosuppression upon detection of sustained BK-viremia for prevention of progression from sustained viremia to BKVAN that may have removed the immunosuppressive selective pressure for mutation of the virus that may have led to progression of disease.

## Figures and Tables

**Table 1:** Basic demographic data of the patients and sample characteristics.

Patient	Viral load^*μ*^			Age	Sex	Type of transplant**	Race	Underlying diagnosis^¶^	Onset of viruria	Peak of viruria^*μ*^	Onset of viremia	Peak of viremia^*μ*^	Mutation*
	Plasma		Urine										
	Sample 1	Sample 2											
1	5.79	5.29	10.24	59	M	DDA	White	DM1, CIN	Week 5	10.35	Week 9	5.79	P(1), D(0), T(3), I(2)
2	5.46	5.32	11.34	76	M	DDA	African American	DM, HTN	Week 9	11.67	Week 13	5.46	P(4), D(1), T(3), I(0)
3	5.85	5.9	11.13	31	M	DDA	White	PSGN, FSGS	Week 6	11.35	Week 7	5.9	P(2), D(0), T(4), I(0)
4	5.24	5.6	8.32	59	M	LRD	White	HTN	Week 1	8.32	Week 5	5.6	P(0), D(1), T(0), I(0)
5	5.35	5.68	8.79	65	M	DDA	White	HTN	Week 2	10.65	Week 13	5.68	P(2), D(0), T(1), I(0)
6	2.5	2.5	12.02	49	M	DDA	African American	DM2, HTN	Week 1	12.02	Week 4	2.5	P(4), D(0), T(2), I(0)
7	5.53	5.26	No sample	59	M	DDA	White	FSGS	Month 9	10.15	Month 9	5.53	P(1), D(0), T(2), I(0)
8	5	2.5	10.1	49	F	DDA	White	MPGN, SLE	Week 3	8.62	Week 5	5	P(2), D(0), T(2), I(1)
9	5.52	5.36	9.65	48	F	LURD	White	IGAN	Week 1	9.65	Week 5	5.52	P(0), D(1), T(0), I(0)
10	5.76	5.17	10.74	23	F	DDA	White	Wegners	Week 3	5.36	Week 7	5.76	P(0), D(0), T(0), I(5)
11	5.76	5.59	10.67	59	M	DDA	White	HTN	Week 1	10.77	Week 4	6.04	P(0), D(0), T(0), I(0)

*I: insertion; P: transversions; D: deletion; T: transitions.

**LRD: living related transplant; DDA: deceased donor allograft, LURD: living un related donor transplant.

*μ*: log_10_

^¶^DM2: type 2 Diabetes Mellitus; HTN: hypertension; FSGS: focal segmental glomerulosclerosis; PSGN: post streptococcal glomerulonephritis; IGAN: IgA Nephropathy; MPGN: membranoproliferative glomerulonephritis; SLE: systemic Lupus Erythematosus.
